# The Impact of Social Isolation on Treatment Burden Among Community‐Dwelling Adults With Disability and Multimorbidity: A Longitudinal Qualitative Study in Urban China

**DOI:** 10.1111/hex.70693

**Published:** 2026-05-12

**Authors:** Jiahui Dong, Ming Yan, Andrew Farmer, Leyi Jiang, Junhai Zhen, Yuling Tong, Yin Dong, Gaofeng Zhang, Lingyan Wu, Yi Guo, Xuejun Yin, Lizheng Fang, Zhijie Xu

**Affiliations:** ^1^ Department of General Practice Sir Run Run Shaw Hospital, Zhejiang University School of Medicine Hangzhou China; ^2^ Department of Emergency Medicine Sir Run Run Shaw Hospital, Zhejiang University School of Medicine Hangzhou China; ^3^ Nuffield Department of Primary Care Health Sciences University of Oxford Oxford UK; ^4^ Department of General Practice, The Second Affiliated Hospital Zhejiang University School of Medicine Hangzhou China; ^5^ Yuhuan People's Hospital Taizhou China; ^6^ School of Public Health, Institute of Biomedical Innovation, Jiangxi Medical College Nanchang University Nanchang China

**Keywords:** depression, disability, longitudinal qualitative study, multimorbidity, social isolation, treatment burden

## Abstract

**Background:**

Social isolation is a critical social determinant of health that amplifies the significant treatment burden faced by community‐dwelling adults with disabilities and multimorbidity. While an association between these factors is established, longitudinal evidence capturing their dynamic interplay is scarce, limiting the development of effective, equitable interventions. This study aimed to longitudinally explore how treatment burden evolves among this population and to elucidate the mechanisms through which social isolation appears to operate through these changes.

**Methods:**

We conducted a longitudinal qualitative study using interpretive description in Hangzhou, China. Participants were adults with physician‐diagnosed disabilities and ≥ 2 chronic conditions, recruited via purposive sampling from community health centres. Each participant completed three in‐depth, semi‐structured interviews over 12 months. We conceptualized treatment burden using Demain et al.'s adaptation of the Cumulative Complexity Model. Data analysis was an iterative process involving constant comparison to identify key themes regarding the interplay of social isolation and treatment burden over time.

**Results:**

A total of 24 participants (13 were women; median age 67.5 years) completed the study. Our analysis revealed that social isolation was described by participants as dynamically contributing to increased treatment burden through four interconnected mechanisms: (1) Eroding autonomy, leading to passive healthcare decision‐making; (2) Compromising emotional well‐being, which depleted self‐management capacity; (3) Straining relational networks, resulting in the loss of crucial informal support; and (4) Creating navigational barriers, which led to difficulties managing complex treatments. A key cross‐cutting theme was the apparent role of depressive symptoms, which participants described as being exacerbated by isolation and, in turn, appearing to contribute to more negative illness perceptions and functional decline. This pattern was consistent with a progressive intensification of treatment burden as emotional and physical challenges fed into each other over time.

**Conclusion:**

Social isolation appeared to function not merely as a passive correlate but as a factor that longitudinally contributed to greater treatment burden, thereby exacerbating health inequities for adults with disabilities and multimorbidity. This pattern appeared to be further shaped by the intersection with depressive symptoms. To mitigate this, multi‐level interventions are essential. Priorities should include addressing structural barriers through policies that foster community integration, strengthening mental health support within primary care, and redesigning services to be more relationally‐centred and less burdensome.

**Patient or Public Contribution:**

Patients, care‐givers, people with lived experience or members of the public were not involved in the study design, conduct, data analysis or preparation of the manuscript. However, preliminary findings were shared and discussed with two patient advisors who had lived experience of disability and multimorbidity but were not participants in the interviews. Their feedback helped refine the presentation and contextual relevance of the themes.

## 1 Introduction

The intersection of disability, multimorbidity and social factors including social isolation represents a growing and critical global health challenge. Disability, defined as long‐term impairment in functioning, affects over 16% of the world's population [1, 2]. A substantial proportion of individuals with disabilities also live with multimorbidity (the coexistence of two or more chronic conditions), creating a complex web of health needs that heightens their risk of premature mortality, functional decline, and diminished quality of life [3, 4, 5]. The relationship is often bidirectional: chronic conditions can precipitate disability, while disability can compound the risk of developing further illnesses [6]. Social isolation, characterized by limited social contacts and engagement, is commonly observed in adults with disabilities and has been linked to worsening health outcomes and mental health challenges [7, 8, 9]. While these associations are well‐documented, the dynamic interplay among disability, multimorbidity, and social isolation in shaping treatment burden, particularly the underlying mechanisms, remains underexplored.

Treatment burden refers to the exhaustive ‘work’ of being a patient and the impact this has on functioning and well‐being [10]. This ‘work’ encompasses not only adhering to medical regimens and attending appointments but also the cognitive, emotional, and social labour of navigating fragmented healthcare systems, coordinating care between multiple specialists, and integrating complex health tasks into daily life [11]. A high treatment burden is robustly linked to poor treatment adherence, adverse health outcomes, and reduced quality of life [12]. For individuals with disabilities and multimorbidity, this burden is often amplified. Physical limitations, chronic pain, and psychological distress (e.g., depression) directly diminish their capacity to manage their health, while the workload imposed by their conditions increases [13]. Systemic barriers, such as fragmented primary care, inadequate social support, and financial constraints, further strain their already limited capacity, leading to an accumulating burden of unmet needs and worsening health disparities [14]. Within this context, existing studies indicate that social isolation is associated with an imbalance between treatment workload and patient capacity.

Recent research has highlighted the complex and bidirectional relationships among social isolation, loneliness, and multiple long‐term conditions (MLTC) [15, 16]. Social isolation, defined objectively as a measurable lack of social contacts and engagement, is not solely a consequence of ill health; in some cases, individuals may actively choose solitude or limited social interaction as a form of self‐protection or preference [17]. It differs from loneliness, which refers to the subjective feeling of being alone. More than a mere consequence of ill health, social isolation is an active contributor to poor outcomes, associated with an increased risk of 32% for all‐cause mortality [18]. It significantly erodes mental well‐being, elevating the risk of depression and anxiety, which in turn depletes the motivation and cognitive resources available for effective self‐management [19]. These patterns suggest the possibility of reinforcing cycles between isolation, mental health, and treatment burden.

While the detrimental impacts of disability, multimorbidity, and social isolation are individually well‐documented, the dynamic interplay through which they collectively shape treatment burden remains poorly understood [20, 21, 22]. Existing research, predominantly cross‐sectional and quantitative, has established a correlation between social isolation and higher treatment burden but fails to elucidate the underlying mechanisms or capture how these experiences evolve over time [23, 24, 25]. Such designs cannot adequately explain how isolation erodes social support networks, how it amplifies emotional distress in the context of daily health management, or how these dynamics shift as a patient's health status changes. To address this critical knowledge gap, specifically the lack of longitudinal qualitative evidence on the underlying mechanisms that link social isolation with treatment burden among adults with both disabilities and multimorbidity, our study employed a longitudinal qualitative design. We therefore aimed to examine how social isolation relates to and influences treatment burden over time in this population. By capturing the lived experiences of community‐dwelling adults with disabilities and multimorbidity over 1 year, we aimed to provide a nuanced, dynamic understanding of how social isolation shapes and intensifies their treatment burden. This research is essential for informing the development of more equitable, person‐centred primary care models that address the social, as well as clinical, needs of this vulnerable population.

## 2 Methods

This study employed a longitudinal qualitative design guided by interpretive description, which is suited for generating relevant knowledge by interpreting participants' experiences within their socio‐contextual reality [26]. A longitudinal approach, involving interviews at three time points over 1 year, was essential to move beyond a static snapshot and capture the dynamic processes through which social isolation influences treatment burden over time. Our reporting adheres to the Consolidated Criteria for Reporting Qualitative Research (COREQ) guidelines [27].

### 2.1 Research Team and Reflexivity

The research team included two primary interviewers: one female (JD) and one male (MY). Both are general practitioners who had no prior clinical relationships with or involvement in the care of any study participants, ensuring that existing therapeutic relationships did not influence participants' responses. Both have formal training in qualitative methodologies. Their clinical background provided an understanding of multimorbidity and the healthcare system, while their training ensured methodological rigour. Both interviewers are fluent in the local dialect spoken by participants, which facilitated effective communication and enhanced rapport during the interviews. The team adopted several reflexive practices throughout the study. Regular debriefing sessions after each interview cycle allowed critical examination of how their clinical assumptions might shape questioning and interpretation. Reflexive memos were maintained to document emerging preconceptions, emotional responses to participants' narratives, and evolving interpretations. The team also engaged in peer debriefing with a social scientist external to clinical practice, which provided alternative perspectives and challenged biomedical framings. Additionally, preliminary findings were discussed with two patient advisors who had lived experience of disabilities and multimorbidity but were not participants in the interviews. This feedback helped strengthen the contextual application and practical resonance of the identified themes.

### 2.2 Setting and Study Population

The study was conducted in five townships in Hangzhou city, China. Participants were adults with disabilities who were registered at a primary care facility serving these townships. We used purposive sampling, guided by the principle of maximum variation, to recruit a diverse sample in terms of age, gender, income, and disability type. Potential participants were identified through the hospital's disability registry and approached face‐to‐face by a researcher.

Patients were included if they were: (1) adults aged 18 years or older; (2) formally diagnosed with two or more chronic conditions, as documented in their medical records; (3) verified by the possession of the official Disability Certificate of the People's Republic of China. Disability types (visual, hearing, physical, intellectual, psychiatric, or multiple disabilities) were determined according to the classification recorded on the certificate; (4) able to communicate in the local dialect or Mandarin Chinese. Exclusion criteria were individuals who were: (1) unable to provide informed consent; (2) in the terminal stage of their illness (expected lifespan < 1 year); (3) planning to move out within the study period.

Recruitment and analysis proceeded iteratively. We initially interviewed 21 participants, at which point thematic saturation was approached. To account for potential attrition and ensure longitudinal data saturation (i.e., adequately capturing patterns of stability and change over time), we recruited five additional participants for a total of 26. Two participants withdrew due to health deterioration, resulting in a final sample of 24 who completed all three interview rounds.

### 2.3 Interview Guide

An interview guide was developed from a comprehensive literature review and drew upon established conceptual frameworks. Questions exploring social networks were informed by the Lubben Social Network Scale [28]; prompts about mood and motivation were adapted from the CES‐D scale [29]; and the exploration of treatment burden was structured around Demain et al.'s [12] framework, covering biographical, relational, and practical aspects. This framework is grounded in the Cumulative Complexity Model, which conceptualizes treatment burden as arising from an imbalance between the workload of healthcare management and a patient's capacity to handle that workload within their personal and social context. The interview guide was originally developed in English and then adapted into Chinese to ensure cultural and linguistic appropriateness. The guide was piloted with three non‐participating individuals and refined based on team discussion. It focused on participants' lived experiences of treatment burden, the perceived social isolation, and the impact of social isolation on treatment burden. The full guide is available in the Supplementary File (see Additional file 1).

### 2.4 Data Collection

Data were collected through in‐depth, semi‐structured interviews at three points: baseline (December 2023), the sixth month (June 2024), and the twelfth month (December 2024). Prior to each interview, the researchers contacted participants to schedule visits at their convenience. Interviews were conducted in Mandarin and the local dialect, depending on participants' preferences, to facilitate effective communication and richer data collection. The research purpose, procedures, and potential risks were explained in detail. Written informed consent was obtained before each interview and after all participants' concerns had been addressed. Participants' demographic data were collected to contextualize the findings. A distress protocol was established prior to data collection. The lead interviewer was trained in psychological first aid and was alert to signs of acute distress. Where significant distress was disclosed, participants were referred to their primary care physician or local community health services, and relevant contact information was provided at the conclusion of each interview. No participants required an acute clinical referral during the study period.

Interviews were conducted in a quiet, private location of the participant's choosing, typically their home. Each interview was planned to last within 1 h. During interviews, researchers explored participants' experiences of treatment burden and their perceived social isolation. A flexible and responsive approach was used, allowing question order and phrasing to be tailored to each participant. Probing questions, such as “Could you tell me more about that” or “Can you give an example,” were frequently used to deepen insights. The interviewers maintained a neutral, non‐judgmental stance to encourage authentic responses. All interviews were audio‐recorded (iFlytek B1), and contextual field notes were taken.

Depressive symptoms were primarily identified through thematic analysis of participants' interview narratives, with attention to self‐reported emotional experiences and descriptions of psychological distress across the three time points. CES‐D scores, administered by trained research staff at each time point as part of a concurrent quantitative component of the broader study, served as a supplementary reference to inform contextual understanding of symptom levels. We note that CES‐D scores reflect screening‐level severity rather than formal clinical diagnosis; references to depressive symptom severity throughout the manuscript therefore reflect patterns emerging from narrative accounts, informed where relevant by corresponding CES‐D scores.

### 2.5 Data Processing and Analysis

All audio recordings were transcribed verbatim into Mandarin within 48 h and checked against the audio for accuracy. To prepare data for analysis and reporting in English, transcripts underwent an iterative process of translation and verification. Initially, a bilingual researcher translated the Mandarin transcripts into English. This translation was then independently back‐translated into Mandarin by a professional linguist. The research team reviewed and reconciled any discrepancies between the original transcripts and the back‐translations to ensure the accuracy and contextual integrity of the final English transcripts used for analysis.

Data analysis was managed using MAXQDA 2020 (VERBI Software, Berlin, Germany) and followed a multi‐stage process. First, two researchers (JD and MY) independently read the transcripts repeatedly and performed open, line‐by‐line coding to capture initial concepts. They met to discuss codes, resolve discrepancies with the participation of a third researcher (ZX), and develop a preliminary codebook. Second, using a constant comparative method, codes were grouped into broader categories. The research team held regular meetings to refine these categories, identify connections, and develop overarching themes and subthemes for the dataset as a whole. The analytical framework for treatment burden themes (biographical disruption, relationship strain, and biological disruption) was informed by the Cumulative Complexity Model as conceptualized by Demain and colleagues [12] in their treatment burden analysis. Themes related to the mechanisms and temporal dynamics of social isolation were identified inductively from the data. In our analysis, we distinguished between depressive symptoms, characterized by participants' own descriptions of persistent low mood, loss of interest, and hopelessness, and the broader intensification of negative emotional states, which encompassed transient or situationally triggered responses such as frustration, anxiety, and loneliness. This distinction was grounded in participants' narrative framing rather than formal clinical assessment, consistent with interpretive descriptive methodology's prioritization of experiential meaning over diagnostic categorization. Third, to specifically address the longitudinal nature of the data, researchers conducted a case‐oriented analysis. For each participant, a summary matrix was created that mapped their experiences, perceptions, and the interplay of social isolation and treatment burden across the three time points. This allowed us to identify individual trajectories of change, stability, or fluctuation. Longitudinal interpretation was constructed through repeated narrative reconstruction across the three interview time points. Temporal sequencing was established analytically by mapping participants' accounts of changes in social connections, emotional distress, depressive symptoms, and treatment burden onto their self‐reported timelines. This involved iterative constant comparison within and across cases, with attention to participants' descriptions of sequence and change over the 12‐month period. These patterns reflect interpretive themes drawn from narrative accounts rather than formal causal or statistical demonstration. It is also important to note that the changes and trajectories described in the findings reflect patterns identified by the research team through thematic analysis across the three interview timepoints, rather than changes explicitly self‐reported by participants at any single timepoint.

In the subsequent cross‐case analysis, trajectories were classified into four broad typologies based on the dominant pattern of change observed across the three time points: (1) progressive worsening, (2) relative stability, (3) fluctuating, and (4) improvement. Not all participants showed intensification over time. While the majority described patterns consistent with progressive worsening, a small number exhibited fluctuating patterns or modest improvement. Contradictions and divergent cases were actively examined through negative case analysis, comparing deviant trajectories with typical ones to refine themes and explore contextual explanations (e.g., differences in socioeconomic resources or informal support). Analytic memos were maintained throughout this process to document key interpretive decisions and ensure a transparent and rigorous analytic trail.

### 2.6 Ethical Considerations

The study was approved by the Ethics Committee of Sir Run Run Shaw Hospital, Zhejiang University School of Medicine. All procedures adhered to the principles of the Declaration of Helsinki [30]. Prior to the first interview, researchers fully explained the study's purpose, procedures, voluntary nature of participation, and confidentiality measures. Written informed consent was obtained from all participants. Capacity to consent was assessed at each interview through brief structured conversation evaluating participants' understanding of the study and their ability to communicate a clear decision. Given the longitudinal design, consent was re‐confirmed at the outset of each subsequent interview to account for potential fluctuation in capacity. To protect anonymity, each participant was assigned a unique code, and all identifying information was removed from transcripts. Participants received a small gift (worth 20 RMB) after each interview to thank them for their time.

## 3 Results

Table 1 outlines the characteristics of the study participants. During the second round of interviews, one participant had died and another declined to participate due to poor health. There were 24 individuals with multimorbidity and disabilities who completed the study. The mean interview duration was 43.5 min (range: 35–56 min). Among the participants, 11 were male and 13 were female, with a median age of 67.5 years (range: 53–81 years). Formally diagnosed chronic conditions included, but were not limited to, hypertension, diabetes, chronic obstructive pulmonary disease, coronary heart disease, and musculoskeletal disorders. Annual household income varied across the sample, with the majority of participants (*n* = 14, 58.3%) reporting incomes below ¥50,000, a threshold reflective of low‐income classifications in the local context. This socioeconomic profile situated the observed patterns within a broader context of disadvantage, where financial constraints often compounded barriers to care, transportation, and social participation. The findings presented below are drawn primarily from interviews with older adults living with physical disabilities and multimorbidity. One participant had an intellectual disability and two had a visual impairment, with no representation of psychiatric disabilities or younger adults. This sample context shaped the patterns described.

**Table 1:** Participant characteristics (*n* = 24).

Variable	*N* (%)
Gender	
Male	11 (45.8%)
Female	13 (54.2%)
Age (years)	
50–59	4 (16.7%)
60–69	11 (45.8%)
70–79	8 (33.3%)
≥ 80	1 (4.2%)
Annual household income (¥)	
< 50,000	14 (58.3%)
50,000–99,999	7 (29.2%)
≥ 100,000	3 (12.5%)
Number of chronic conditions	
2	2 (8.3%)
3	12 (50%)
4	8 (33.3%)
5	2 (8.3%)
Type of disability	
Physical disability	21 (87.5%)
Visual impairment	2 (8.3%)
Intellectual disability	1 (4.2%)

### 3.1 Treatment Burden Experience

#### 3.1.1 Biographical Disruption

Multimorbidity and disability disrupted participants' sense of identity and continuity in life. Many described a gradual loss of independence and increasing reliance on family members to access healthcare. Dietary restrictions conflicted with family routines and reduced autonomy. Participants often felt transformed from capable individuals to burdens and experienced diminished self‐worth. Some expressed that needing personal care felt intrusive and compromised their privacy and dignity.

I can't walk anymore… Even going to the hospital for my medication has become a challenge. I have to rely on my daughter, and I always feel like I'm dragging her down and causing trouble for the family.

(P001)

Consistent with Demain et al.'s [12] treatment burden framework, in which biographical disruption includes negative affective states (e.g., anxiety, helplessness, and depression) as consequences of long‐term treatment demands, many participants expressed emotional distress linked to prolonged illness and limited treatment efficacy. When therapy failed to meet expectations, they often reported anxiety, helplessness, and depression, which is commonly observed among those undergoing long‐term care. A few also described a sense of social stigma related to their disability, further exacerbating their emotional burden.

I often feel heartbroken. I know this disease is incurable. Even if I went out it wouldn't be cured. It's like running a race where I can slow down but never stop. I feel overwhelmed by worry about being alone and losing my family.

(P012)

A perceived loss of purpose was also prevalent. Many reported being unable to engage in once‐meaningful activities such as farming, singing, or physical work, due to physical decline. Some noted that they could no longer contribute to the household in familiar ways that intensified their sense of loss and frustration.

Before I got sick I worked in the fields from dawn to dusk. Now even walking a few steps to the garden to tend vegetables has become difficult. I can't do anything anymore.

(P009)

#### 3.1.2 Relationship Strain

Many participants with multimorbidity and disabilities reported difficulties stemming from the complexity of treatment. Conflicts sometimes emerged when participants preferred more conservative approaches while family members advocated for more aggressive options. The disagreements strained family relationships and created additional burden. The need for long‐term caregiving placed emotional and practical stress on family members, friends, and neighbours. As treatment demands intensified, some participants became aware of their dependence on others and described themselves as burdens or even ‘parasites’ on their caregivers. This sense of guilt was a source of internalized distress.

Of course I feel guilty. It's inevitable right? Anyone with a conscience would feel the same. My wife is healthy and complete and I rely entirely on her. I'm like a parasite.

(P012)

Several participants reported a shrinking of their social networks as a result of illness, leading to feelings of isolation and being neglected. Reduced social engagement was often attributed to mobility limitations, perceived stigma, or lack of external support that may further contribute to poor psychological and physical health.

We don't really interact with the people nearby. When they walk past our door I can feel their contempt. They look down on us, on disabled people.

(P006)

#### 3.1.3 Biological Disruption

Physical discomfort and long‐term medication use were frequently mentioned as important experiences of treatment burden. Some participants described persistent side effects such as nausea, abdominal pain, or dizziness, particularly from analgesics or neuroprotective agents. These symptoms contributed to reduced medication adherence and growing scepticism toward pharmacological treatments. Others reported disrupted sleep and cognitive impairment, which they attributed to continued drug use.

I have to keep taking these pills but the more I take the worse I feel…My mind is slipping. I don't really want to take them anymore. I can't sleep even by 2 a.m.; my nerves feel overwhelmed. I told my kids but there's not much they can do.

(P015)

### 3.2 Social Isolation

#### 3.2.1 Barriers to Social Interaction

Some participants attributed their isolation to practical barriers in maintaining social contact. The conditions such as leg weakness, visual impairments and dizziness restricted their mobility. Distance from relatives and unfamiliarity with digital communication, especially among older adults from rural areas, further reduced their ability to stay connected with others.

It's hard for me to go out. If something happens to my brain vessels I could collapse outside. The doctor told me to stay home. When I go out my heart races and I feel nervous and my blood pressure shoots up.

(P015)

I can't see my phone screen clearly. I'm using an old‐style mobile and I never had schooling so I don't know how to use a smartphone. It's just too difficult for me.

(P016)

#### 3.2.2 Self‐Imposed Withdrawal

Psychological factors also contributed to social isolation. Some participants deliberately withdrew from social contact, citing feelings of rejection and low self‐worth. They described reducing interactions because they felt unable to offer help and worried about burdening others. Many turned to watching TV for entertainment and grew accustomed to solitude, losing interest in socializing. Others felt excluded from group activities that did not accommodate their disabilities, which further discouraged participation.

I used to make lots of phone calls and chat with people all the time. But not anymore. Now I just scroll through news on my phone.

(P002)

I don't want to go out with them. I can barely care for myself so why trouble them? I'm afraid of getting lost or collapsing in the street. I've heard about stories where people who tried to help someone were wrongly blamed.

(P014)

#### 3.2.3 Restrictive Environment

In some cases, social isolation was shaped by environmental limitations. Some participants expressed a strong desire for social interaction but reported a lack of community activities. The absence of organized events left them without channels for engagement and increased their sense of disappointment and helplessness.

In our community, there are no group activities. I'd like to go out and see things but there's never anything happening.

(P004)

Participants also highlighted the lack of disability‐friendly infrastructure, particularly for those using wheelchairs. The absence of accessible roads or toilets restricted their physical movement and made it difficult to participate in social activities.

I can't walk and have to use a wheelchair. It's inconvenient to get around in a wheelchair. The paths outside are full of stairs that I can't navigate and even going to the toilet is a problem.

(P017)

These environmental restrictions functioned as relatively stable structural barriers across the three interview waves (baseline, 6 months, and 12 months). Their core features remained consistent, while their influence on social participation persisted throughout the study period, contributing to the overall picture of isolation factors.

### 3.3 Impact of Social Isolation on Treatment Burden Experience

#### 3.3.1 Diminished Independence and Autonomy

Participants described prolonged social isolation as accompanied by a reduced sense of autonomy and increased dependence on others for daily tasks and treatment decisions. Some participants could manage their routine activities like preparing meals early on, but as conditions worsened, essential tasks such as bathing required caregiver assistance. Limited support deepened feelings of helplessness, with personal needs often overlooked. Social isolation was also described in connection with a diminished role in treatment decisions, which were often made by caregivers due to mobility limitations. Participants reported lacking confidence to express concerns and complied passively with decisions made on their behalf.

I used to enjoy going to the senior centre to sit and chat while others played cards. Now my son won't let me go because he says I'm too ill. So, I just stay home watching TV.

(P008)

My family insisted on taking me to a certain hospital, but I didn't like the place. I thought the doctors were too young and inexperienced. I wasn't happy but no one changed the plan.

(P017)

**Figure 1:**
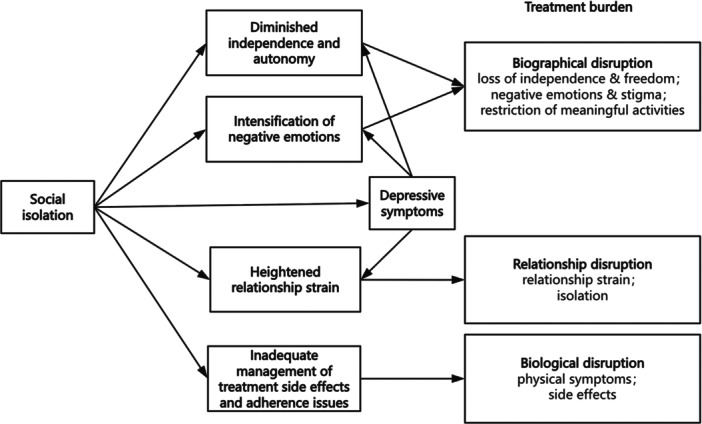
Pathways through which social isolation and depressive symptoms influence the experience of treatment burden. Social isolation is shown in relation to diminished independence, intensification of negative emotions, depressive symptoms, heightened relationship strain, and inadequate management of treatment side effects and adherence issues. These experiences connect to biographical disruption, relationship disruption, and biological disruption. Arrows represent conceptual associations and dynamic interrelationships identified through participants' narratives; they do not imply unidirectional causation or formal statistical mediation.

Limited access to information fostered hopelessness and disengagement. Participants described feelings consistent with depressive symptoms, including helplessness and low motivation, in connection with reduced self‐management. Over time, this led to heightened treatment burden, manifesting as increased reliance on others and restrictions on meaningful activities that perpetuated biographical disruption (Figure 1). For instance, one participant described how initial mobility barriers from isolation led to emotional withdrawal:

At first being stuck at home because I couldn't easily visit friends made me feel cut off. But as the months went on it turned into this deep sadness where I didn't even want to try managing my own appointments anymore. I just let my family decide everything.

(P005)

Another participant expressed the repeated sense of hopelessness that compounded this disengagement: ‘*I've had this illness for years and it hasn't improved. It torments me but I can't beat it and I don't think anyone can…Nobody could help me beat it’. (P002)*

#### 3.3.2 Intensification of Negative Emotions

Social isolation was described as exacerbating participants' emotional distress, manifesting as persistent feelings of anxiety and depression. While some emotional responses in this theme may resemble features of depression, participants consistently framed them as reactive and situational rather than pervasive or enduring, which informed our analytic separation from the depressive symptoms discussed above. This intensification often evolved into a chronic state where the lack of meaningful social connections amplified participants' reported emotional distress. Participants described reduced interpersonal interactions as contributing to feelings of disconnection and a cycle of rumination on health uncertainties.

My relatives don't really care much about me; they only visit once or twice a year. Every day I just stay alone with nothing to do, and when I'm alone it's easy to keep thinking about things, like whether my illness will keep getting worse.

(P011)

I don't talk to anyone…I can't go out because it's so depressing. I just sit here all day with nothing I want to do.

(P010)

Ongoing isolation worsened uncertainty about health and recovery trajectories. Participants reported that their confidence in self‐managing conditions eroded over time, leading to heightened fear and doubt about their health status. This was compounded by financial hardships, in which context isolation limited opportunities for informal support or income‐generating activities, amplified emotional distress and created a pervasive sense of despair. Such experience was observed across interviews, with many noting a progressive deepening of negative emotions as isolation persisted.

I used to run a small repair shop but business has dropped. Fewer people come by now even to chat. I can't make a living from it anymore. At first I just felt worried about money but now it's become this constant sadness. I sit alone in the empty shop all day with no one to talk to.

(P004)

These patterns appeared more pronounced among participants facing socioeconomic disadvantages. Many described how limited financial resources restricted their ability to access transportation, pay for caregiving support, or participate in community activities, thereby intensifying both social isolation and the daily demands of treatment management. This intersection created additional layers of emotional and practical burden.

For me, the biggest problem is money. If I could afford to hire someone to take care of me, my family wouldn't be so exhausted, and I wouldn't feel so miserable. The good medicine for this illness is also very expensive, so I can't afford the better ones. Not to mention going out; leaving home costs even more money, and the pressure on me is enormous.

(P012)

Participants described what they experienced as depressive symptoms in this process. These symptoms such as persistent low mood, loss of interest in activities, and fatigue were frequently described by participants as exacerbating their emotional burden and reducing motivation for treatment adherence and self‐care. Participants who described more severe depressive symptoms during initial interviews seemed to experience more pronounced emotional escalation. Many recounted patterns consistent with isolation contributing to depressive feelings, which in turn seemed to reinforce withdrawal and emotional deterioration, reflecting the accumulating imbalance between workload and capacity.

At first it was just a bit of helplessness when my condition worsened and I couldn't get out much. But without anyone around to talk to or help during those bad days it turned into full depression. I'd cry alone, lose sleep, and feel like giving up on everything.

(P007)

#### 3.3.3 Heightened Relationship Strain

Participants described how social isolation strained relationships with family members, healthcare providers, and broader support networks by reducing opportunities for interaction and contributing to feelings of being burdensome or unsupported. This relational disruption appeared linked to depressive symptoms such as guilt and emotional withdrawal. Their accounts often reflected patterns consistent with a conceptual cycle in which deteriorated relationships and isolation reinforced each other over the 12 months (as shown in Figure 1). This pattern seemed to contribute to increased treatment burden through reduced informal support and heightened emotional demands. We note that these observations represent conceptual interpretations drawn from narrative accounts rather than statistical mediation analysis.

Participants expressed a sense of guilt about burdening their children and disappointment over limited companionship. These tensions intensified over time as family contact remained infrequent and emotional needs continued to go unmet. Some participants further internalized these strains by withholding their needs because they feared imposing additional stress on their relatives.

My son lives far away. One day I felt like I was having a stroke and called him to say goodbye. He cried and called an ambulance. Since then he calls every night and visits monthly. It's hard for both of us.

(P013)

When I feel unwell, I just bear it. I don't tell my children because they won't take me to the doctor so I don't bother.

(P005)

In interactions with healthcare providers, social isolation widened the communication gap. As contact with the outside world decreased, participants found it harder to describe symptoms or understand medical advice, which increased their reluctance to seek care.

I can tell the doctor what hurts, but I don't understand much of what they say. I can't explain things well, so I need my grandson to go with me.

(P008)

Furthermore, in broader social networks, some participants maintained limited social ties with neighbours, but these weakened over time due to reduced mobility. Some even experienced social rejection due to illness‐related hygiene issues.

Before I fell, I could still move around. Now, I'm bedridden. Neighbours used to visit but now they say I smell of urine and don't want to come.

(P019)

#### 3.3.4 Inadequate Management of Treatment Side Effects and Adherence Issues

Our findings suggested that social isolation impeded timely access to care and left side effects unmanaged. Participants described various obstacles preventing them from addressing medication side effects promptly. Travel difficulties including lack of accessible transportation and the absence of escorts made clinic visits burdensome and sometimes impossible. Over time side effects worsened or became normalized as part of daily experience. This delay was further described in connection with cognitive impairments and low health literacy, which limited participants' ability to recognize when symptoms warranted professional attention.

This medication upsets my stomach but I haven't gone to the hospital. It's too much trouble so I just endure it.

(P021)

Reduced clinic attendance was described as relating to delays in medication adjustments and appeared linked to the risk of medication misuse and adverse events. Isolation from healthcare providers meant that patients had no reliable source for clarifying doubts about medication use or learning about safer alternatives. This knowledge gap was particularly evident among participants who turned to traditional remedies or non‐prescribed treatments.

I didn't know I shouldn't use moxibustion on the same spot for too long… I ended up with burns on my leg.

(P004)

Social isolation also contributed to medication non‐adherence, a problem that appeared to worsen over the follow‐up period. Memory impairments were reported as a common cause, particularly among older participants. Participants frequently forgot to take medications or became confused about usage and dosage if a little reminder was given from their caregivers. This pattern was further complicated by complex polypharmacy regimens involving multiple medications with different timing requirements.

My daughter reminds me to take my pills…When she's not here I would forget it…One afternoon I got dizzy and realized I hadn't taken my blood pressure medicine.

(P023)

## 4 Discussion

This longitudinal qualitative study explored how social isolation related to treatment burden among community‐dwelling adults with disabilities and multimorbidity. Through 12‐month follow‐up with three interview waves, participants' accounts suggested that social isolation acted as a pervasive factor contributing to increased treatment burden across biographical, relational, and biological dimensions. Our findings suggest that the relationship between social isolation and treatment burden appeared bidirectional rather than strictly linear, with social isolation operating through interconnected pathways that seemed to build over time and depressive symptoms serving as one element linking social disconnection to challenges in self‐management capacity. These patterns were most pronounced among participants facing socioeconomic disadvantage, highlighting how social isolation perpetuates health inequities within already vulnerable populations.

Our findings align with biographical disruption concepts in chronic illness research, where participants described social isolation as progressively contributing to the erosion of autonomy and identity. Bury's seminal work conceptualized biographical disruption as the profound interruption of taken‐for‐granted assumptions caused by chronic illness, a phenomenon extensively documented in multimorbidity populations where disease fragments sense of self [31, 32]. Our longitudinal data extend this understanding by revealing patterns consistent with isolation contributing to these disruptions over time. Participants who initially managed basic self‐care later required extensive assistance as isolation limited informal support and eroded confidence. Early mobility barriers evolved into chronic withdrawal while initial family tensions deepened into entrenched relational breakdowns. This temporal progression addresses a gap in systematic reviews that called for investigations into treatment burden evolution rather than static snapshots [12, 33]. The apparent accumulating effects of sustained isolation highlight the value of incorporating temporal dynamics into treatment burden frameworks, particularly for populations facing intersecting vulnerabilities.

Social isolation in our study stemmed from three interconnected drivers: physical barriers to mobility, psychological withdrawal, and environmental limitations. Physical barriers such as mobility impairments, visual deficits, and cardiovascular symptoms restricted participants' ability to leave home and engage with others, consistent with evidence that functional limitations reduce social participation among disabled populations [18, 34]. Our findings reveal how psychological factors, including fear of burdening others, low self‐worth, and anticipated social rejection compelled self‐imposed withdrawal even when physical access existed. Participants deliberately reduced social contact and became accustomed to solitude despite desiring connection, patterns consistent with research on felt stigma among disabled populations [35]. Environmental limitations further constrained participation as community infrastructure lacked disability‐friendly features such as accessible pathways and toilets while organized social activities were absent [36]. Over the study period, these barriers accumulated and intensified. Participants who initially maintained limited social ties experienced progressive network constriction as physical decline worsened, psychological resignation deepened, and environmental barriers remained unaddressed. These environmental and structural constraints align with evidence on disability infrastructure deficits in resource‐limited settings, where inaccessible transportation and limited community facilities restrict social participation among adults with physical disabilities [37]. In the Chinese context, heavy reliance on family caregiving reflects filial piety norms, which may discourage formal service use and heighten burden when family resources are limited [38]. Such cultural and infrastructural factors appeared to interact with individual‐level isolation, contributing to the observed variability in treatment burden experiences.

Our longitudinal data reveal patterns consistent with social isolation appearing to contribute to increased treatment burden through interrelated processes that seemed to build over time. Research links social disconnection to reduced medication adherence, higher hospitalization rates, and greater symptom severity [39]. Our study demonstrates specific pathways: diminished autonomy in self‐management, inadequate management of medication side effects, and delayed care‐seeking due to lack of escorts or informational support. These mechanisms align with treatment burden frameworks conceptualizing social resources as critical capacities offsetting illness management work [40]. Participants' accounts included what seemed like a cycle in which early isolation appeared to reduce capacity to manage treatment demands, relating to symptom escalation and further withdrawal. These experiences align with established models showing how cascading failures and accumulating vulnerabilities create widening disparities among individuals with disabilities over time [41]. This temporal progression underscores the urgency of addressing social isolation as a structural inequity requiring population‐level intervention. The majority of participants in our study reported annual household incomes below ¥50,000, situating their experiences within a broader context of socioeconomic disadvantage where financial constraints further limited access to paid care, transportation, and social activities. These patterns suggest that the compounding vulnerabilities documented in this study are not solely products of individual illness trajectories, but also reflect systemic gaps in community‐based support and disability welfare provisions that disproportionately affect older adults with physical disabilities and multimorbidity in resource‐limited settings.

Depressive symptoms emerged as a critical pathway through which social isolation appeared linked to amplified treatment burden in our study. Depression is associated with relationships between social isolation and adverse health outcomes, including reduced self‐care and poor medication adherence among multimorbid individuals [42, 43]. Our findings illustrate how this pathway operated over time. Participants who reported more pronounced depressive experiences early in the study described a progressive deepening of emotional distress across the 12 months. This appeared to generate a reinforcing cycle, in which isolation‐related distress promoted further withdrawal and diminished self‐management capacity. This pattern aligns with longitudinal evidence showing depression both results from and perpetuates social isolation [44]. Depression eroded autonomy and agency among our participants as they exhibited reduced decision‐making confidence and passive treatment compliance, consistent with evidence that depression undermines self‐management in chronic illness [45]. Critically, depressive symptom persistence was shaped by a lack of formal mental health services as participants rarely accessed psychological support and normalized untreated depression, reflecting systemic inequities in mental health care access for disabled and disadvantaged populations [46].

Our findings highlight the need for multilevel interventions addressing the interconnected pathways between social isolation, depression, and treatment burden identified in this study. Social prescribing programs that connect patients to peer support groups and community activities show promise for reducing loneliness and improving mental health when adapted to local contexts [47]. Within health systems, integrating mental health care into chronic disease management through collaborative care models has demonstrated effectiveness for people with multimorbidity [48]. At the policy level, government support through disability allowances and caregiver subsidies can improve access to care for disabled populations [49]. Interpreted through a clinical lens, these findings suggest that social isolation may function as a compounding factor in treatment burden. Future research should investigate how clinicians currently consider social isolation within routine practice, for example, by exploring the feasibility of incorporating brief social network assessments alongside standard clinical evaluations. Studies could also examine approaches to concurrently addressing co‐occurring depressive symptoms and social disconnection, such as through referrals to peer support or simplification of treatment regimens for patients with limited informal support. Such investigations would provide actionable insights for more equitable, person‐centred care. Future research should employ mixed‐methods designs to validate the temporal relationships documented in this qualitative study and evaluate intervention impacts across subgroups defined by socioeconomic position and disability severity to provide actionable guidance for equitable policy and practice.

This study has several limitations. First, recruitment from a single urban district may restrict generalisability, though maximum variation sampling ensured diversity in demographics and clinical profiles; additionally, the sample was relatively homogeneous in terms of disability type and participant characteristics: it consisted primarily of older adults with physical disabilities and multimorbidity. Consequently, the findings may not be generalizable to broader disabled populations. Second, many participants had rural backgrounds and low education levels, potentially limiting response depth; this was mitigated by using simple, jargon‐free language. Third, capturing and interpreting non‐verbal cues proved challenging due to cultural and individual variations, addressed via real‐time notes and post‐interview debriefs with staff to triangulate observations. Fourth, the 1‐year timeframe may have constrained observations of long‐term changes, mitigated by selecting recently registered participants to capture early shifts. Finally, given the complexity of patients' health conditions and the presence of multiple confounding factors, we limited the follow‐up period to minimize bias introduced by time‐varying influences.

## 5 Conclusion

This longitudinal study demonstrates that social isolation is a major determinant in the development of treatment burden among adults with disabilities and multimorbidity. Operating through a self‐reinforcing cycle linked to depressive symptoms, isolation erodes biographical identity, depletes self‐management capacity, and ultimately entrenches the health inequities that health systems aim to solve. Addressing this challenge requires a fundamental shift beyond purely clinical interventions. To mitigate the impact of social isolation on health, policy and practice should address the broader social and structural determinants by promoting community integration, enhancing access to mental health support within primary care settings, and redesigning healthcare services to actively foster strong relationships and meaningful social connections at all levels of care.

## Author Contributions

**Jiahui Dong:** conceptualization, data curation, formal analysis, investigation, methodology, validation, writing – original draft, writing – review and editing. **Ming Yan:** conceptualization, investigation, data curation, formal analysis, investigation, methodology, writing – original draft, writing – review and editing. **Andrew Farmer:** formal analysis, methodology, supervision, writing – review and editing. **Leyi Jiang:** formal analysis, methodology, writing – review and editing. **Junhai Zhen:** data curation, methodology, writing – review and editing. **Yuling Tong:** conceptualization, investigation, formal analysis, writing – review and editing. **Yin Dong:** investigation, methodology, writing – review and editing. **Gaofeng Zhang:** data curation, methodology, writing – review and editing. **Lingyan Wu:** methodology, writing – review and editing. **Yi Guo:** validation, writing – review and editing. **Xuejun Yin:** conceptualization, methodology, supervision, writing – review and editing. **Lizheng Fang:** conceptualization, resources, funding acquisition, writing – review and editing. **Zhijie Xu:** conceptualization, validation, funding acquisition, supervision, writing – review and editing.

## Ethics Statement

Ethical approval for the study was obtained from the Sir Run Run Shaw Hospital, Zhejiang University School of Medicine (Hospital Ethics Review Approval 2024 Research No. 0216).

## Consent

All participants gave written informed consent.

## Conflicts of Interest

The authors declare no conflicts of interest.

## Supporting information

Supporting File.

## Data Availability

The participants of this study did not give written consent for their data to be shared publicly, so due to the sensitive nature of the research, supporting data is not available.
