# Supplementary material for: The Impact of Social Isolation on Treatment Burden Among Community‐Dwelling Adults With Disability and Multimorbidity: A Longitudinal Qualitative Study in Urban China
Source: Health Expect. 2026 May 12;29(3):e70693. doi: 10.1111/hex.70693 (PMC13163148; doi:10.1111/hex.70693)
Supplement: Supplementary file 1 — Supporting File. [file HEX-29-e70693-s001.docx]

**Supplement file** Interview Guide

**Initial interview (baseline)**

***Part 1: Opening and Introduction***

Dear participant,

Thank you very much for taking the time to participate in this interview. This is a study about the experiences of managing chronic conditions and disabilities. We hope that by listening to your experiences and feelings, we can better understand the challenges and needs you face in managing your health, with the ultimate aim of helping to improve healthcare services and support systems in the future. The content of this interview will be kept strictly confidential and used for academic research purposes only. Your participation is entirely voluntary. You may skip any question or choose to withdraw from the interview at any time without this affecting your access to any healthcare services or entitlements. The interview is expected to last approximately 40-60 minutes.

Before we begin, do you have any questions? If you are comfortable to proceed, I would like to start by requesting your consent to participate and to audio-record our conversation for accurate transcription. Are you willing to participate under these conditions?

***Part 2: Questions***

Section A: Understanding Health Situation

1. To start, could you tell me in your own words about your current health? What are the main conditions or health issues you are dealing with?

-Besides this, do you have any other ongoing or long-term health conditions?

1. Could you describe the disability or the main difficulties with daily activities that you experience? How long have you been living with this?
2. Overall, how would you sum up your experience of managing these health conditions so far? What has that journey been like for you?

Section B: Experiences with Healthcare and Management

4. Walking me through a typical day or week, how do you usually manage your health conditions? (For example, what routines or tasks do you follow?)

(If applicable) If you keep track of things like your blood pressure or sugar levels, how do you do that? What is that process like for you?

(If applicable) Could you tell me about your experiences with taking medications? This could include remembering to take them, getting prescriptions, side effects, or costs.

5. How does dealing with your healthcare (e.g., appointments, medications, or therapies) affect your finances or your need for daily practical support?

6. How does managing your health impact your everyday life and your relationships with people around you?

-Could you give me an example of when someone's help made a real difference? Or an example of a challenge you faced because you didn't have the support you needed?

-When dealing with this gets tough and your mood dips, how do you usually handle those feelings?

7. What feels most challenging or overwhelming about keeping on top of your health?

8. Could you describe what it's like for you when you see your doctors or go for healthcare visits?

-Think about things like getting to the clinic, talking with the doctors or nurses, or any difficulties you might have during these visits.

9. Is there anything else about your treatments or healthcare routine that you'd like to share, that we haven't touched on yet?

Section C: Emotional Well-being

10. Many people in similar situations experience periods of sadness, frustration, or feel discouraged. Have you felt this way? If so, what usually contributes to these feelings, and how often do they occur?

11. Do you ever worry about being a burden to your family or friends because of your health needs? Why or why not?

12. Have you noticed any changes in what you're interested in, how you think, or your ability to concentrate? Can you give me an example?

13. How have your eating habits and sleeping patterns been lately?

14. How does your mood or emotional state affect your daily life and your ability to manage your health? Any examples come to mind?

15. Is there anything else about your mood or emotional well-being that you feel is important for me to understand?

Section D: Social Connections and Support

16. To help me understand your daily life, who do you live with? And who are the people you interact with most regularly?

17. How do you usually stay in touch with your relatives (like children, siblings, or other family not living with you)? How often do you have contact?

18. What about friends or neighbours? How often do you connect with them?

19. How often do you get out of the house to go somewhere or participate in any community or social activities?

20. Do you sometimes feel disconnected from others or that you lack companionship? If so, could you tell me more about what that feels like?

21. Thinking about managing your health—things like taking medications, going to appointments, or daily tasks—how do you feel limited social contact or support affects your ability to handle these? (For better or for worse).

22. Is there anything else on the topics of your social life, support, or community connections that you'd like to add?

***Part 3: Closing***

Thank you so much for sharing your experiences and thoughts with me today. Your insights are incredibly valuable and will contribute greatly to our understanding. All the information you have provided will be handled confidentially. Do you have any final questions for me about the study or about what we discussed today?

Finally, as this is a longitudinal study, I would like to confirm if you are willing for us to contact you again for a follow-up conversation in a few months’ time. Would that be alright?

Thank you once again for your time and your important contribution!

**Follow-up interview (month 6 & month 12)**

***Part 1: Opening and Introduction***

Hello [Participant’s Name]! It's good to see you again. First of all, thank you so much for continuing to participate in our study. It’s been about six months since we last spoke, and I’m very interested to hear how you’ve been doing during this time. Just as before, everything we discuss will be kept strictly confidential and used only for research purposes. You are still free to skip any question or pause the interview at any time. Today's conversation should also last about 45-60 minutes. Before we begin, do you have any questions about the process?

If not, let’s get started. I'd like to begin by asking about your health.

***Part 2: Core Questions***

Section A. Changes in Health Conditions

1. Overall, how has your health been since we last spoke? Have you noticed any new issues, or perhaps some improvements in certain areas?
2. Have there been any changes in the difficulties you experience with daily activities, or in any of your chronic conditions?

-What do you think might have led to these changes?

1. Compared to six months ago, how would you say the overall management of your health conditions is going now?

Section B. Evolving Experiences of Treatment Burden

4. Looking back, how has the actual work of managing your conditions changed since our last interview?

-For example, have there been any shifts in how you monitor your health or in your medication routines?

-What do you think brought about those changes?

5. Has there been any change in how your healthcare needs affect your finances or in the kind of practical support you receive from others?

6. Lately, how has managing your health been impacting your daily life and your interactions with people? Does it feel different from before?

-Can you think of any new examples, either of helpful support you've received, new challenges you've faced, or how it's been affecting your mood?

-What were the reasons behind these situations?

1. Over the past few months, what new challenges in managing your health have come up?

-Have any previous difficulties gotten better or resolved? Why do you think that happened?

1. How have your interactions with doctors or other healthcare providers been?

-Have you noticed any changes in getting appointments, communicating with them, or understanding your care plan?

9. Is there anything else about changes in your treatment or healthcare routine that you feel is important to share?

Section C. Changes of Emotional Well-being

10. Have your feelings (e.g., sadness, discouragement, or being hard on yourself) changed in any way over the last six months?

-What might explain these shifts, if they happened?

11. Has there been any change in how much you worry about being a burden to others? Why do you think that is?

12. What about your interest in things, your ability to focus, or your general thought patterns, have you noticed any differences?

-Could you give me an example and what might be influencing it?

1. Have your eating habits or sleeping patterns shifted?

-What do you feel could be influencing that?

1. Compared to before, how has your mood or emotional state been affecting your daily routines and your ability to stick with health tasks lately?
2. Is there anything else about changes in your emotional well-being that you’d like to mention?

Section D. Changes of Social Connections and Their Impact

16. Have there been any changes in your living arrangements or in the people you interact with most on a daily basis?

17. Has there been a shift in how often or how you connect with your relatives (like children, siblings, or other family)?

-What are the reasons for this change in contact or closeness?

18. What about your connections with friends or neighbours?

19. How has getting out of the house or participating in community or social activities been going?

20. Have your feelings of being disconnected from others, or of having companionship, changed at all? Please tell me more about why you think that is.

21. Thinking about now compared to six months ago, how is the level of social support you have (or the lack of it) affecting your ability to manage your conditions differently?

-For instance, are the impacts on taking medications, monitoring your health, or daily tasks different? What's causing that?

-Has it affected getting to or communicating during doctor’s visits in a new way?

22. Is there anything else you’d like to add about changes in your social life or support?

***Part 3: Closing***

Thank you once again for such a thoughtful and detailed conversation. It is incredibly helpful for me to understand how things have evolved for you over these months. Is there any important experience or feeling from the past six months that we haven't covered today, but that you feel is central to your experience of managing your health and well-being?

I truly appreciate your ongoing participation. Your insights are shaping our understanding in very meaningful ways. (If applicable: As planned, we will be in touch again for a final conversation in another six months.) Please don’t hesitate to contact me if you have any questions before then.

Thank you, and I wish you all the best until we speak again!
